# Enhancing Nonylphenol Biodegradation: The Role of Acetyl-CoA C-Acetyltransferase in *Bacillus cereus*

**DOI:** 10.3390/biotech14040099

**Published:** 2025-12-18

**Authors:** Fanglian Lu, Deqin Luo, Lian Yang, Ranran Dong

**Affiliations:** College of Animal Science, Guizhou University, Guiyang 550000, China; gs.fllu23@gzu.edu.cn (F.L.); gs.dqluo24@gzu.edu.cn (D.L.); gs.lianyang22@gzu.edu.cn (L.Y.)

**Keywords:** *Bacillus cereus*, acetyl-CoA C-acyltransferase (AtoB), nonylphenol (NP), lipidomics, gene overexpress, bacterial degradation

## Abstract

Nonylphenol (NP) bioremediation is constrained by the scarcity of efficient and non-pathogenic degrading strains. To clarify the role of acetyl-CoA C-acetyltransferase (AtoB) in NP degradation, we generated an *atoB*-overexpressed strain (LY-OE) from the environmentally tolerant *Bacillus cereus* LY and compared its degradation rate with the wild type using HPLC. Untargeted lipidomics was conducted to characterize metabolic responses under NP stress, and key differential lipid metabolites (DELMs) were further validated by ELISA. Additionally, AtoB concentration and ATP content were quantified using commercial assay kits in *Bacillus cereus*. LY-OE showed a markedly higher NP degradation rate (96%) than LY (85%). Lipidomic analysis identified 34 significant DELMs (VIP > 1, *p* < 0.05), including elevated cardiolipin (CL) and phosphatidylglycerol (PG), and reduced phosphatidylcholine (PC) and triglycerides (TG). ELISA confirmed these changes (*p* < 0.01 or *p* < 0.001), consistent with lipidomic findings. LY-OE showed significantly higher AtoB concentration during the logarithmic growth phase and exhibited higher ATP content during NP degradation. These findings suggest that *atoB* overexpression enhances NP degradation by both boosting energy supply and remodeling lipid metabolism. This work identifies *atoB* as a key factor for NP biodegradation and provides a promising strategy for developing high-performance bioremediation strains.

## 1. Introduction

As a typical environmental endocrine disruptor (EDC), nonylphenol (NP) exhibits notable chemical stability, hydrophobicity, and bioaccumulation [1,2]. NP in the environment mainly comes from the incomplete degradation of nonylphenol ethoxylates (NPEOs), nonionic surfactants from industrial and domestic wastewater. NP is widely present in aquatic environments, sediments, and soils. It is likely to be transmitted through the food chain, thereby presenting a substantial risk to ecosystems and human health [3,4,5]. NP has the capacity to mimic estrogen and interacts with the endocrine systems of mammals and aquatic organisms, even at exceedingly low concentrations (ng/L level). These interactions can exert a notable impact on their immune systems, reproductive development, and a variety of other physiological aspects [6,7]. Furthermore, it has been implicated in the onset and progression of certain cancers [8,9] and has been classified as a priority pollutant by several countries. The three primary environmental remediation methods for NP are chemical oxidation, biodegradation, and physical adsorption. Although physicochemical approaches are characterized by their rapidity, they often face significant challenges, including elevated treatment costs, the risk of secondary pollution, and difficulties associated with large-scale implementation [10,11]. Conversely, biodegradation technology presents a more promising alternative due to its sustainability, cost-effectiveness, and environmental compatibility. NP functioned as a carbon and energy source by microorganisms, particularly bacteria, and subsequently underwent enzymatic transformation into compounds with reduced toxicity or were progressively mineralized into carbon dioxide and water [12].

The seminal study on NP biodegradation was published in 1999, documenting the ability of *Sphingomonas* sp. strain TTNP3 to degrade NP [13]. Subsequently, numerous studies have unanimously demonstrated that strains within the same genus, such as *Sphingomonas cloacae* [14] and *Sphingomonas xenophaga* [15], are capable of degrading NP. In the current research on NP biodegradation, the bacterial genus *Pseudomonas* has been extensively investigated [16]. However, other genera capable of degrading NP include *Aspergillus* [17], *Citrobacter* [18], *Clostridium* [19], and *Pseudomonas* [20]. Nevertheless, the majority of these documented NP-degrading bacteria are either pathogenic or conditionally pathogenic. Because of its exceptional resistance to various environmental conditions, including heat, dryness, chemical poisons, and radiation [21], *Bacillus cereus* has been extensively used as a probiotic owing to its functions in promoting host growth, metabolism, and microecological balance [22]. Interestingly, *Bacillus* also demonstrated a strong capacity for pollutant degradation, including the enzymatic breakdown of several toxins [23] and the effective breakdown of high phenol concentrations [24]. Our previous research also demonstrated that *Bacillus cereus* LY has strong biodegradation ability against NP (under review). Acetyl-CoA C-acyltransferase is an important enzyme in the initial stages of the alkane/phenol degradation pathway [25]. Studies have confirmed this enzyme’s activity in the tropical pseudohyphae peroxisome, linked to alkane metabolism, and recent research has elucidated its role in the degradation of phenolic compounds [26]. Research findings indicate that under oligotrophic conditions, the expression of acetyl-CoA C-acyltransferase in the phenol degradation pathway of *Rhodococcus erythropolis* B403 is significantly upregulated [27]. This implies that acetyl-CoA C-acyltransferase may not only play a direct role in the metabolism of phenols but may also act as a source of energy and carbon by regulating fatty acid production. This regulation could potentially enhance the degradation process and improve bacteria’s resistance to environmental stress.

In summary, we speculate that acetyl-CoA C-acetyltransferase plays a crucial role in the pathway of NP degradation in *Bacillus cereus*. Our study focuses on the impact of the acetyl-CoA C-acetyltransferase gene *atoB* in *Bacillus cereus* LY on the degradation efficiency of NP and detected the differential metabolites. We also detected the AtoB concentration and the ATP content in *Bacillus cereus*. Furthermore, we combined the lipidomic with the previous genomic data of *Bacillus cereus* (CRA021210) in our research group to analyze the pathway of atoB degrading NP. These findings are expected to provide essential theoretical support for the targeted engineering of *B. cereus* LY and would offer a basis for its potential application in environmental remediation.

## 2. Materials and Methods

### 2.1. Chemicals, Strains, and Growth Conditions

The analytical standard nonylphenol (NP, C_15_H_24_O, purity 93.8%, molecular weight 220.35 g/mol, CAS: 84852-15-3) was purchased from Sigma-Aldrich (St. Louis, MO, USA).

The *Bacillus cereus* LY strain utilized in this experiment was originally isolated from catfish, preserved in our laboratory, and subsequently screened and acclimated for the efficient degradation of NP. *B. cereus* LY was cultivated at 37 °C and 150 rpm in a shaking incubator using TSB liquid medium containing 1 mg/L of NP.

### 2.2. Construction of the Bacillus cereus atoB Overexpression Vector

The *atoB* gene fragment was amplified by PCR using *Bacillus cereus* LY DNA genome as a template and the primers atoB-pHT01-F/atoB-pHT01-R. Simultaneously, the pHT01 plasmid was used as a template with the primers pHT01-atoB-F/pHT01-atoB-R to generate linearized vector fragments by reverse PCR amplification. After purification of the amplification product, the *atoB* gene fragment was ligated via seamless cloning to the linearized pHT01 vector. The ligated product was transformed into *E. coli* DH5α competent cells, plated onto an ampicillin-resistant agar plate, and incubated at 37 °C until the OD_600_ reached 0.6. After that, induction was performed by adding IPTG, and the culture was incubated overnight. Positive clones were identified by colony PCR. All the PCR products were subjected to agarose gel (1%) electrophoresis, and the gels were stained with ethidium bromide to visualize bands. The *Bacillus cereus* LY *atoB* gene overexpression vector was obtained by screening and sequencing the positive clones. Table 1 displays the primer sequences.

### 2.3. Quantification of AtoB by ELISA

The concentration of the AtoB (acetyl-CoA C-acetyltransferase) was determined using a commercial Enzyme-Linked Immunosorbent Assay (ELISA) kit (Shanghai Yuanju Biotechnology Center, Shanghai, China) in accordance with the manufacturer’s protocol. Bacterial cultures in the mid-logarithmic phase were collected by centrifugation. The bacterial pellets were resuspended in extraction buffer and subjected to sonication on ice (200 W, 3 s pulse, 10 s interval, 30 cycles). The lysate was then centrifuged at 12,000× *g* for 10 min at 4 °C. The supernatant was collected and immediately placed on ice. AtoB concentration was calculated by interpolation from a standard curve generated in parallel. Three biological replicates were obtained for each group, with each replicate being measured in triplicate.

### 2.4. Biodegradation of NP

The wild-type strain LY (control) and the overexpression strain LY-OE (experimental) were inoculated together into a medium supplemented with 1 mg/L TSB for the NP degradation assay. Three biological replicates were set up per group. Before the experiment, all experimental materials and components were sterilized via autoclaving or ultraviolet (UV) irradiation according to their suitability. Each 250 mL flask was supplemented with 100 μL of NP stock solution (1 g·L^−1^ in methanol), evaporated for 24 h, then supplemented with 100 mL TSB medium to achieve 1 mg·L^−1^ NP. Bacterial suspension was inoculated at an initial concentration of approximately 10^8^ CFU·mL^−1^.

The samples were incubated at 37 °C with shaking at 150 rpm for 7 days. Then, 1.0 mL of each sample was transferred to a 15 mL centrifuge tube, mixed with 10 mL of n-hexane, vortexed for 1 min, ultrasonicated for 10 min, and centrifuged at 9560× *g*/min for 4 min. The resulting supernatant was collected and stored for subsequent analysis.

### 2.5. Detection of NP by HPLC

The sample was processed using solid–liquid extraction. An activated carbon column (SPE column, the column was packed with polystyrene-divinylbenzene (HLB)) was placed under gravity flow. 2 mL of n-hexane was added to precondition the column, followed by loading 3 mL of sample extract. Then, the column was washed with 2 mL of n-hexane and then eluted with 2 mL of acetone. The eluate was dried under a stream of nitrogen gas at room temperature and reconstituted with 1 mL of a methanol-water mixture (80:20, *v*/*v*) and filtered through a 0.22 μm membrane into a sample vial for subsequent analysis.

The concentration of NP was determined using high-performance liquid chromatography (HPLC, Waters 2695, Waters Corporation, Milford, MA, USA). The analytical conditions were as follows: a C18 reverse-phase column (250 mm × 4.6 mm, 5 μm), mobile phase composed of methanol: water (89:11, *v*/*v*), flow rate of 0.5 mL/min, column temperature maintained at room temperature, injection volume of 10 μL, and detection wavelength set at 225 nm. NP standard solutions (31.25, 62.5, 125, 250, 500, 1000 μg/mL) were prepared in n-hexane. Upon injection, peak areas were recorded, and a calibration curve was constructed by plotting NP standard concentrations (*x*-axis) against peak areas (*y*-axis). Linear regression analysis was then performed with a significance level of α = 0.05 (*p* < 0.05). These experimental procedures were carried out in accordance with the methodology detailed by Cai [28]. Statistical analysis was performed using GraphPad Prism 9.5, and intergroup differences were assessed using an independent samples *t*-test (*p* < 0.05).

### 2.6. Measurement of ATP Content in Bacillus cereus

The ATP content in both wild-type and recombinant *Bacillus cereus* strains was measured using a commercial ATP assay kit (Beijing Boxbio Science & Technology Co., Ltd., Beijing, China) according to the manufacturer’s instructions. The strains were cultured in NP-containing medium. Bacterial samples were collected by centrifugation on days 1, 3, 5, and 7 of the NP degradation process.

The bacterial pellets were resuspended in extraction buffer and disrupted by sonication on ice (200 W, 3 s pulse, 10 s interval, 30 cycles). The lysates were then centrifuged at 10,000× *g* for 10 min at 4 °C. A 500 μL aliquot of the supernatant was mixed thoroughly with 500 μL of chloroformand centrifuged at 10,000× *g* for 5 min at 4 °C. Finally, the upper aqueous phase was collected and maintained on ice for ATP assay. The ATP concentration in each sample was calculated by interpolating from a standard curve run in parallel.

### 2.7. Lipid Metabolome Detection

The control and experimental groups (for specific operations, see Section 2.3) were cultured in a constant-temperature shaker at 37 °C with shaking at 150 rpm for 7 days. After incubation, all samples were transferred to 50 mL centrifuge tubes and centrifuged at 4 °C, 13,000× *g* for 15 min. The supernatant was discarded, and the pellets were stored for subsequent use.

Fifty milligrams (50 mg) of the sample were accurately weighed into a 2 mL tube containing a 6 mm grinding bead. Then, 280 μL of an internal standard-containing lipid extraction solution (methanol: water = 2:5, *v*/*v*) and 400 μL of methyl tert-butyl ether (MTBE) were added. The mixture was homogenized at −10 °C and 50 Hz for 6 min, followed by ultrasonic extraction at 5 °C and 40 kHz for 30 min, and subsequent incubation at −20 °C for 30 min. The resulting solution was centrifuged at 4 °C and 13,000× *g* for 15 min. A 350 μL aliquot of the supernatant was transferred, dried under a stream of nitrogen, and reconstituted with 100 μL of isopropanol: acetonitrile (1:1, *v*/*v*). The reconstituted solution was vortexed for 30 s, subjected to sonication at 5 °C and 40 kHz for 5 min, and then centrifuged at 4 °C and 13,000× *g* for 10 min. Finally, the supernatant was collected for subsequent analysis.

Lipid analysis was performed using a UHPLC-Q Exactive HF-X system (Vanquish, Thermo Fisher Scientific, Waltham, MA, USA). A Accucore C30 column (100 mm × 2.1 mm, 2.6 µm) was used for chromatographic separation. Mobile phase A consists of 50% acetonitrile in water (containing 0.1% formic acid and 10 mmol/L ammonium acetate), and mobile phase B consists of acetonitrile: isopropanol: water (10:88:2, *v*/*v*/*v*) containing 0.02% formic acid and 2 mmol/L ammonium acetate. The column temperature was maintained at 40 °C, and the injection volume was 3 µL. Electrospray ionization was conducted in both positive and negative ion modes. Quality control (QC) samples were prepared by pooling equal volumes of lipid extracts from each sample, followed by centrifugation.

### 2.8. Lipid Qualitative and Quantitative Analyses

LipidSearch software (Version 4.0) (Thermo Fisher, Waltham, MA, USA) [29,30,31] was used for data processing and lipid identification by matching MS and MS/MS spectra to a metabolite database, with a mass error threshold under 10 ppm and identification based on MS/MS scores. Quantification was based on the area under the curve (AUC) for each lipid. Following normalization and removal of duplicates, the data underwent log-transform and were analyzed with orthogonal partial least squares discriminant analysis (OPLS-DA) using the ropls R package [29,32] (Version 1.6.2). Differentially expressed lipids were identified with variable importance in projection (VIP) values > 1 and *p* < 0.05 from independent sample *t*-tests [29,32,33].

### 2.9. ELISA Validation of Differential Lipids

Enzyme-linked immunosorbent assay (ELISA) was used to detect four significantly different lipids (cardiolipin (CL), phosphatidylglycerol (PG), phosphatidylcholine (PC), triacylglycerol (TG)). Specifically, the CL, PG, PC, and TG kits were purchased from Beijing Jingmeike Biotechnology Co., Ltd. (Beijing, China), Shanghai Kanglang Biotechnology Co., Ltd. (Shanghai, China), Wuhan Elabscience Biotechnology Co., Ltd. (Wuhan, China), and Beijing Boxbio Science & Technology Co., Ltd. (Beijing, China), respectively. CL, PG, PC, and TG measurements were detected according to the manufacturer’s instructions. The standard curve was generated using Excel to calculate the sample concentrations. Statistical analysis refers to Section 2.4.

### 2.10. Statistical Analysis

The results were presented as mean ± standard deviation (*n* = 3). Statistical analysis was performed using GraphPad Prism 9.5, and intergroup differences were assessed using an independent samples *t*-test (* *p* < 0.05; ** *p* < 0.01, *** *p* < 0.01).

## 3. Results

### 3.1. PCR Identification of atoB Overexpression in Bacillus cereus LY

**Figure 1:**
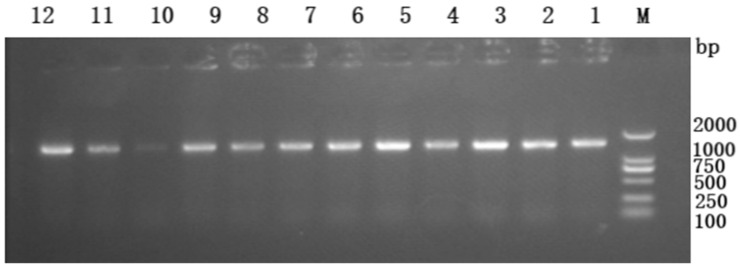
PCR identification of LY-atoB-PHT01 overexpression colony. Numbers 1–12 in the figure indicate the 12 randomly selected clone numbers.

After PCR amplification, 1% agarose gel electrophoresis analysis showed a clear target band around 1700 bp (Figure 1). Sequencing results confirmed the fragment was from *Bacillus cereus* LY and exhibited 99.82% identity with the target gene *atoB*, indicating successful cloning.

### 3.2. Acetyl-CoA C-Acetyltransferase Concentrations

**Figure 2:**
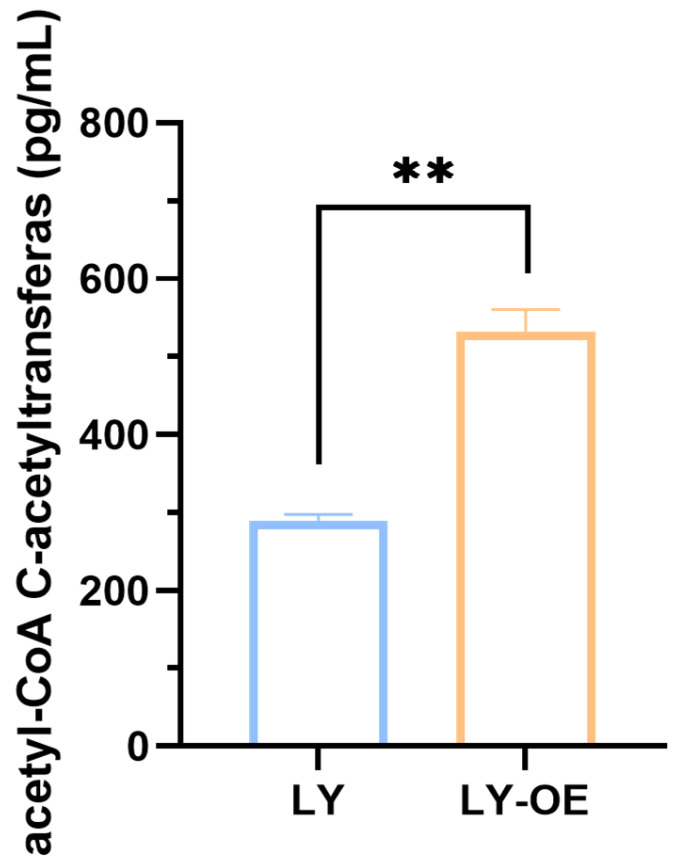
AtoB concentration in wild-type (LY) and *atoB*-overexpressed (LY-OE) *Bacillus cereus* strains, ** *p* < 0.01.

Quantitative analysis revealed that the AtoB concentration in the LY-OE strain (531.57 ± 28.51 pg/mL) was significantly greater than that in the LY strain (289.42 ± 8.00 pg/mL; *p* < 0.001) (Figure 2). This result confirmed the successful overexpression of the *atoB* gene, and consequently, a significant increase in AtoB protein levels in the recombinant *Bacillus cereus*.

### 3.3. Overexpression of the atoB Gene in Bacillus cereus Enhances Its Degradation Rate of NP

**Figure 3:**
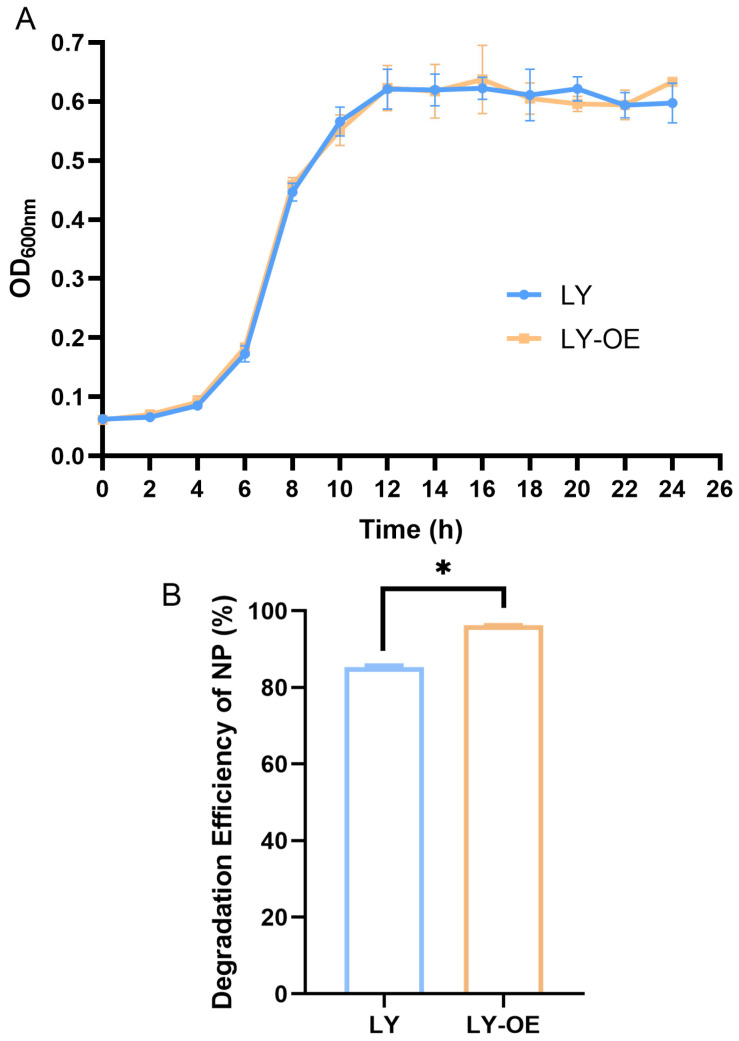
Growth curves and NP degradation experiments. (**A**) Growth curves of wild-type strain LY and overexpression strain LY-OE. (**B**) NP degradation efficiency of bacterial strains LY and LY-OE after 7 days of incubation, * *p* < 0.05.

The growth curves of LY-OE strain and LY strain showed no obvious difference, indicating that *atoB* does not affect the normal growth of LY-OE strain (Figure 3A).

The wild-type LY strain degraded NP by 85% after 7 days of incubation, while LY-OE degraded it by an average of 96% (Figure 3B and Figure S1–S3). The degradation efficiency of the LY-OE group increased by 11%, which was significantly higher than that of the wild-type LY group.

### 3.4. ATP Content in Bacillus cereus During NP Degradation

**Figure 4:**
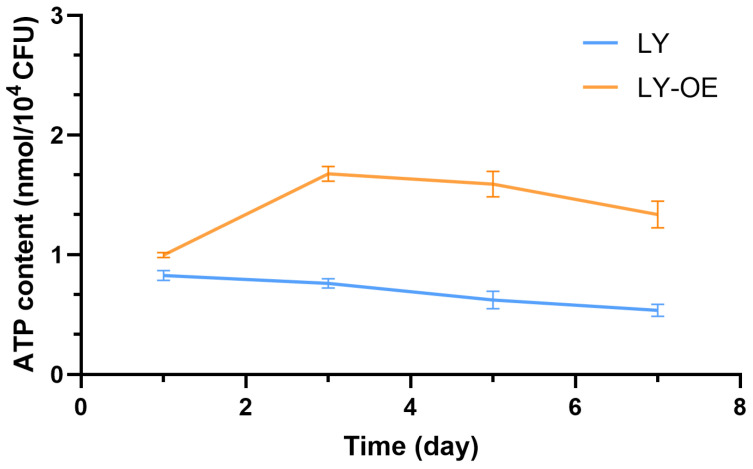
ATP content in wild-type (LY) and *atoB*-overexpressing (LY-OE) *Bacillus cereus* strains under NP stress over time.

Under NP stress, the ATP content in the wild-type (LY) strain decreased significantly and remained at a low level. In contrast, the *atoB*-overexpressing strain (LY-OE) exhibited a remarkable increase in ATP content, reaching a peak on day 3 at approximately 1.68 ± 0.06 nmol/10^4^ CFU, a level twofold higher than that in the NP stressed LY strain (*p* < 0.01) (Figure 4). Although declining thereafter, the ATP level in LY-OE remained significantly higher than in LY throughout the degradation process.

### 3.5. Lipid Metabolomics Analysis

**Figure 5:**
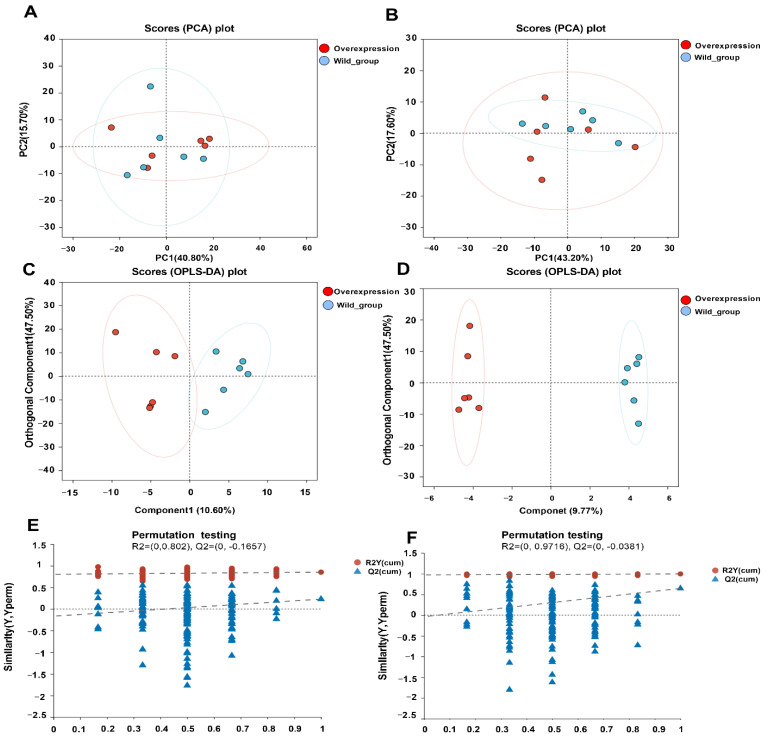
Multivariate statistical analysis. (**A**) Plot of PCA scores in positive ion mode. (**B**) Plot of PCA scores in negative ion mode. (**C**) Plot of OPLS-DA scores in positive ion mode. (**D**) Plot of OPLS-DA scores in negative ion mode. (**E**) Validation of the OPLS-DA model in positive-ion mode. (**F**) Validation of the OPLS-DA model in negative ion mode.

Principal component analysis (PCA) was performed in unsupervised mode to assess the clustering of samples. Results showed strong clustering within groups, indicating reliable data. The PCA score plots (Figure 5A,B) revealed significant spatial separation between the wild-type and *atoB* overexpression groups, suggesting that *atoB* gene overexpression may influence NP degradation in *Bacillus cereus*. Orthogonal partial least squares discriminant analysis (OPLS-DA) was used to further resolve the differences between groups. The resulting OPLS-DA plots (Figure 5C,D) showed that the two samples were separated and that the classification effect was significant, indicating that the strains’ metabolic characteristics were globally reconstructed as a result of the overexpression of the *atoB* gene. With R^2^X = 0.581, R^2^Y = 0.852, and Q^2^ = 0.228 in the positive-ion model (Figure 5E) and R^2^X = 0.751, R^2^Y = 0.995, and Q^2^ = 0.645 in the negative-ion model (Figure 5F), the robustness of the OPLS-DA model was confirmed by 200 permutation tests, demonstrating the goodness of fit (R^2^X and R^2^Y) and predictability (Q^2^).

**Figure 6:**
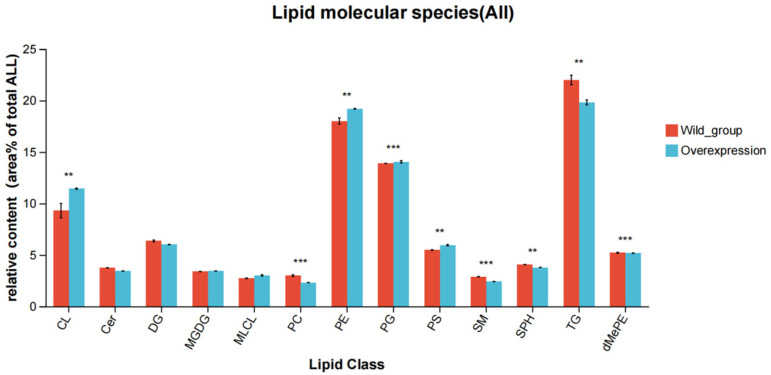
Bar chart for classification of lipids. Horizontal coordinates are the different lipid subclasses, vertical coordinates are the sum of the contents of different subgroups of the same lipid class in the middle, ** *p* < 0.01, *** *p* < 0.001.

**Figure 7:**
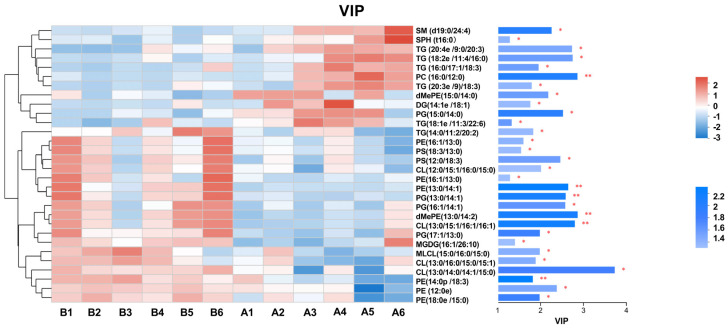
VIP analysis diagram. The left side is the metabolite cluster tree chart, and the right side is the metabolite VIP bar chart. A1–A6 are the wild-type strain, and B1-B6 are the *atoB* overexpressing strain. The bar length represents the contribution value of the metabolite to the difference between the two groups, * *p* < 0.05, ** *p* < 0.01.

Differential lipid metabolite analysis: based on the orthogonal partial least squares discriminant analysis (OPLS-DA) model, intergroup differences in lipid metabolic profiles between the wild-type strain and the *atoB*-overexpressing strain were analyzed. Using variable importance in projection (VIP) values > 1 and *t*-test *p* < 0.05 as dual thresholds, a total of 34 significantly differential lipid metabolites (DELMs) were identified, which were established as potential biomarkers of gene overexpression effects, mainly including glycerophospholipids (GP), sphingolipids (SP), and glycerolipids (GL) (Table 2). The lipid classification bar chart revealed that cardiolipin (CL), phosphatidylglycerol (PG), and phosphatidylethanolamine (PE) were significantly upregulated, whereas phosphatidylcholine (PC) and triacylglycerol (TG) were significantly downregulated (Figure 6). Based on the identified significant DELMs, a heatmap was generated using hierarchical clustering analysis, integrated with a VIP bar plot for comprehensive visualization (Figure 7). This analysis intuitively revealed the divergent expression trends of DELMs between the wild-type and *atoB*-overexpressing strains, as well as their contribution strengths to intergroup differences. Results indicated that the overexpression and wild-type groups formed distinct clustering branches, with CL, PC, PE, PG, and TG contributing most significantly to the observed group differences.

### 3.6. ELISA Validation Results

**Figure 8:**
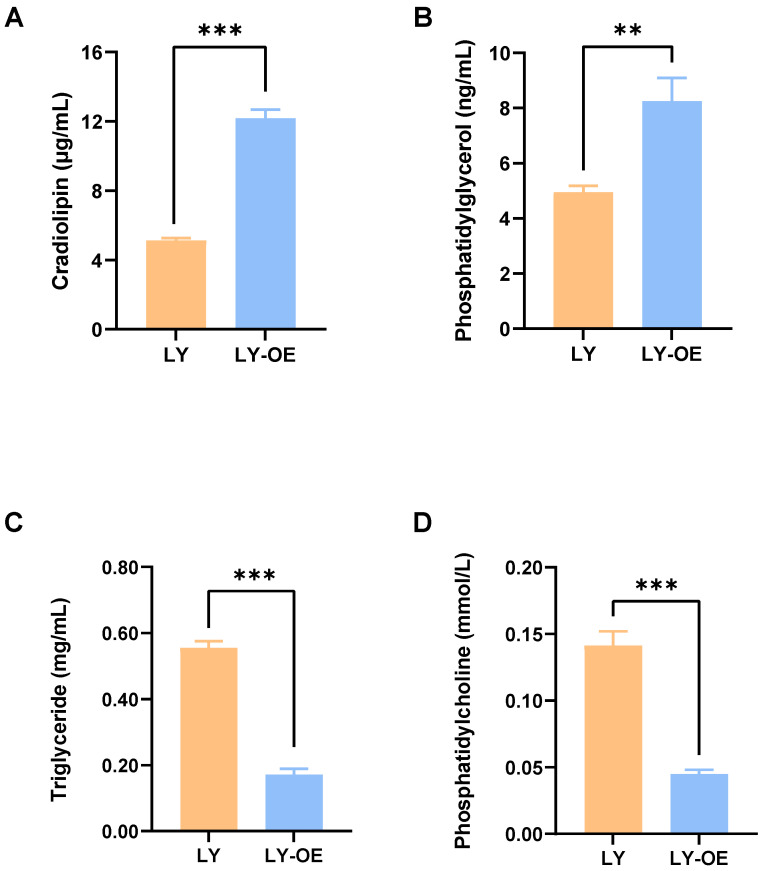
Validation results of differential lipids by ELISA. (**A**) Cardiolipin (CL) content in bacterial strains LY and LY-OE; (**B**)Phosphatidylglycerol (PG) content in bacterial strains LY and LY-OE; (**C**) Triglyceride (TG) content in bacterial strains LY and LY-OE; (**D**) Phosphatidylcholine (PC) content in bacterial strains LY and LY-OE. Significant differences between groups are indicated with asterisks. ** *p* < 0.01, *** *p* < 0.001.

To verify the reliability of the lipid metabolomics results, we used an ELISA kit to detect the concentrations of four differential lipids, CL, PG, PC, and TG, in *Bacillus cereus* LY after degradation of NP. The ELISA results showed that, compared with the wild-type strain LY, the levels of CL and PG in the *atoB* overexpressing strain LY-OE were significantly higher (*p* < 0.01), and the levels of PC, TG were significantly lower (*p* < 0.001) (Figure 8). The experimental results showed that the ELISA validation results were consistent with the lipid metabolomics results.

## 4. Discussion

Recent studies focus on eco-friendly microbial pathways, using genetic engineering to boost bacterial enzyme activity for better pollutant degradation. For instance, genetically enhanced bacterial extracellular electron transfer (EET) significantly boosts bioremediation of methyl orange (MO) and hexavalent chromium (Cr (VI)) [34]. Similarly, cloning and expressing the laccase gene via vectors enhances its activity, accelerating the degradation of dye pollutants like bromophenol blue [35]. Moreover, promoter modifications have been shown to elevate laccase and peroxidase expression, prominently improving ciprofloxacin degradation [36]. In comparison, research on genetically engineering organisms to degrade NP remains limited. Recently, CRISPR-modified *Pseudoxanthomonas mexicana* showed improved chemotaxis and NP degradation [37], marking progress in this field. In this study, we found that overexpressing *atoB* in *Bacillus cereus* enhanced NP degradation. This finding identifies *atoB* as a new genetic engineering target for bioremediation, though the underlying mechanism requires further elucidation.

Our lipidomic analysis revealed that *atoB* overexpression induced significant alterations of membrane lipids in *Bacillus cereus*, characterized by a significant increase in phosphatidylethanolamine (PE), phosphatidylglycerol (PG), and cardiolipin (CL), alongside a marked decrease in triacylglycerol (TG). Based on these observations, we propose that this coordinated remodeling may constitute an adaptive strategy whereby the bacterium simultaneously enhances its energy supply and optimizes membrane function to facilitate NP degradation. A pivotal change was the upregulation of CL, a key phospholipid in the bacterial cytoplasmic membrane known for its role in stabilizing the electron transport chain (ETC) complexes [38,39]. The increased CL abundance could potentially enhance respiratory efficiency and ATP production, which is crucial for the energy-demanding process of NP degradation. Our ATP content measurement support this hypothesis by showing a significant increase specifically in the *atoB*-overexpressed strain under NP stress, confirming its enhanced energy status. Notably, the biosynthesis of CL in bacteria is directly linked to its precursor, PG, through a condensation reaction catalyzed by cardiolipin synthase [38]. Consequently, the observed upregulation of PG is intrinsically linked to the elevated CL levels, forming a coherent metabolic response aimed at enhancing energy transduction. Beyond enhancing energy supply, the parallel increase in PE and PG suggests a sophisticated remodeling of membrane architecture and function. We suppose that the upregulation of PE, a non-bilayer-prone lipid, promotes the formation of non-lamellar phases [40,41], thereby facilitating membrane fusion and vesicles transport in *Bacillus cereus*. Concurrently, we hypothesize that the synchronized upregulation of PE and PG synergistically optimizes membrane functionality. On one hand, PG can form a dense hydrogen-bond network with PE, which enhances membrane tightness and reduces nonspecific permeability, thereby limiting the passive leakage of toxic NP intermediates [42,43]. This lipid remodeling could, in principle, serve to limit the passive leakage of toxic NP intermediates. On the other hand, its role likely extends beyond simply “solidify” the membrane. Substantial evidence indicates that membrane lipid composition, particularly non-bilayer lipids like PE and anionic lipids like PG, can directly regulate the activity and conformation of membrane transport proteins [44,45]. A canonical example is that the function of certain transport systems is strictly dependent on the presence of PE [46]. Therefore, we hypothesize is that the altered lipid environment in the LY-OE strain serves a dual role: as a selective barrier and as a facilitator for the efficient transport of NP or its metabolites, thereby ensuring effective substrate uptake.

Beyond membrane remodeling, the significant decrease in TG indicates a systemic metabolic shift towards energy mobilization. As the primary energy storage lipid, the decrease in TG suggests that strain LY-OE is actively catabolizing lipid reserves to meet the high energy demand of NP degradation. TG breakdown provides fatty acids for β-oxidation, generating additional acetyl-CoA, reducing equivalents (NADH/FADH2), and ATP [47]. The higher AtoB concentration observed in our study, along with the decreased TG and increased ATP, indicates that stored energy is efficiently mobilized and converted into energy for NP degradation. This finding aligns with previous reports that overexpression of thiolase family members can suppress TG biosynthesis [48], and it is consistent with the role of atoB in acyl transfer and facilitating energy release during metabolism.

**Figure 9:**
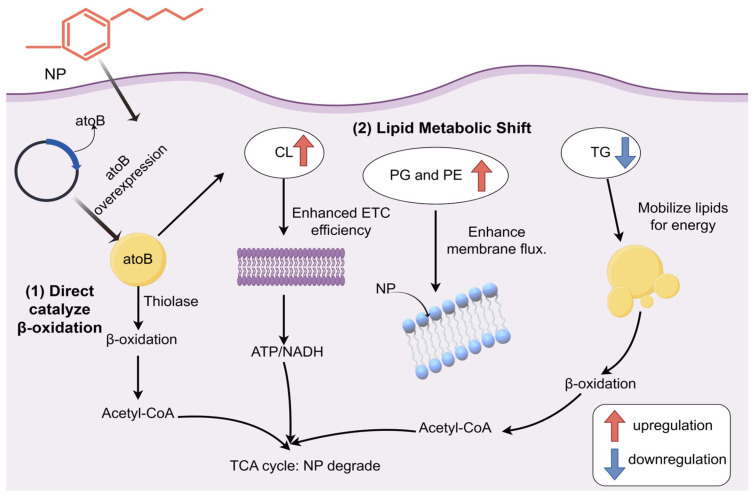
Diagram of the NP proposed degradation pathway. The proposed degradation pathway figure was generated using Figdraw 2.0.

In summary, combining the existing research and our experimental data (AtoB concentration and ATP content), and the data of the *Bacillus cereus* genome (CRA021210) we previously obtained, we postulate that the overexpression of *atoB* enhances NP degradation by *Bacillus cereus* via two distinct pathways (Figure 9): (1) directly improving β-oxidation cycle to accelerate side chain cleavage, and (2) triggering lipid metabolism reprogramming. This reprogramming includes upregulating CL to enhance the electron transport chain (ETC) efficiency and downregulating TG to mobilize lipid reserves, thereby ensuring a sufficient supply of ATP and reducing power. Synergistically regulating membrane phospholipids (PE↑/PG↑) optimizes membrane transport function. Together, these changes construct an efficient degradation network that significantly enhances the strain’s capability to handle NP.

Finally, while our study links *atoB* overexpression to enhance NP degradation, the complete catabolic pathway remains to be mapped. To this end, future work will employ 13C-labeled NP to precisely trace the degradation flux, identify key intermediates and final products, and elucidate the full biodegradation process.

## 5. Conclusions

This study demonstrates that overexpressing the *atoB* gene in *Bacillus cereus* enhances the degradation efficiency of NP. This enhancement is achieved through its influence on metabolite levels, such as CL, PG, PC, and TG. The coordinated regulation of these lipids enhances energy supply and optimizes membrane function, leading to a marked acceleration in NP degradation. Therefore, *atoB* functions not only as a degradative enzyme but also as a pivotal global regulator of lipid metabolism to facilitate NP removal. Our finding thus provides precise targets and strategies for engineering high-performance microbial strains for NP bioremediation. Future research could focus on essential DELMs, such as the cardiolipin synthase gene (*cls*) and the dtriglyceride hydrolase genes, in this degradation process.

## Figures and Tables

**Table 1:** Primer information.

Primer Name	Sequence Information
atoB-pHT01-F	TTAAAGGAGGAAGGATCCATGAATAGAGCAGTCATTGTAG
atoB-pHT01-R	GTGGTGGTGGTGGTGGTGGTCTTCTATTTTCTCAAATAAT
pHT01-atoB-F	CACCACCACCACCACTAGCAGCCCGCCTAATGAGCGGGCTT
pHT01-atoB-R	ATGACTGCTCTATTCATGGGATCCTTCCTCCTTTAATTGGGG
pHT01-JD-F	CCGGAATTAGCTTGGGTACCAGCT
pHT01-JD-R	AACCATTTGTTCCAGGTAAGG

**Table 2:** Differential metabolites and their trends.

Metabolites	Classification	Molecular Formula	VIP	P	FC	Trend
SPH (t16:0)	SP	C_16_H_36_O_3_N_1_	1.2863	0.0385	0.9719	↓
Cer (t16:1/17:0)	SP	C_33_H_66_O_4_N_1_	1.1613	0.0378	0.9610	↓
SM (d19:0/24:4)	SP	C_48_H_92_O_6_N_2_P_1_	2.2581	0.0108	0.8757	↓
dMePE (15:0/14:0)	GP	C_36_H_71_O_8_N_1_P_1_	2.1811	0.0265	0.8456	↓
dMePE (13:0/14:2)	GP	C_34_H_63_O_8_N_1_P_1_	2.8655	0.0088	1.3143	↑
PE (16:1/13:0)	GP	C_34_H_65_O_8_N_1_P_1_	1.6028	0.0377	1.0674	↑
PE (13:0/14:1)	GP	C_32_H_61_O_8_N_1_P_1_	2.6455	0.0061	1.2369	↑
PE (18:0e/15:0)	GP	C_38_H_78_O_7_N_1_P_1_K_1_	1.9791	0.0148	1.0931	↑
PE (16:1/14:3)	GP	C_35_H_62_O_8_N_1_P_1_Na_1_	1.2907	0.0463	1.1681	↑
PE (12:0e/18:1)	GP	C_35_H_71_O_7_N_1_P_1_	2.3793	0.0315	1.1171	↑
PE (14:0p/18:3)	GP	C_37_H_69_O_7_N_1_P_1_	1.8208	0.0058	1.0704	↑
PS (18:3/13:0)	GP	C_37_H_65_O_10_N_1_P_1_	1.5490	0.0445	1.0717	↑
PS (12:0/18:3)	GP	C_36_H_63_O_10_N_1_P_1_	2.4624	0.0270	1.2339	↑
MLCL (15:0/16:0/15:0)	GP	C_55_H_107_O_16_P_2_	1.9832	0.0360	1.1546	↑
CL (12:0/15:1/16:0/15:0)	GP	C_67_H_126_O_17_P_2_	2.0132	0.0471	1.1443	↑
CL (13:0/14:0/14:1/15:0)	GP	C_65_H_122_O_17_P_2_	3.7290	0.0168	1.8013	↑
CL (13:0/15:1/16:1/16:1)	GP	C_69_H_126_O_17_P_2_	2.7992	0.0075	1.3056	↑
CL (13:0/16:0/15:0/15:1)	GP	C_68_H_129_O_17_P_2_	1.8872	0.0338	1.1319	↑
PC (16:0/12:0)	GP	C_36_H_73_O_8_N_1_P_1_	2.8606	0.0099	0.8072	↓
PG (16:1/14:1)	GP	C_36_H_66_O_10_N_0_P_1_	2.5766	0.0231	1.2690	↑
PG (13:0/13:0)	GP	C_32_H_62_O_10_N_0_P_1_	1.1192	0.0439	0.9572	↓
PG (13:0/14:1)	GP	C_33_H_62_O_10_N_0_P_1_	2.5864	0.0084	1.2704	↑
PG (17:1/13:0)	GP	C_36_H_68_O_10_N_0_P_1_	1.9851	0.0154	1.1062	↑
PG (15:0/14:0)	GP	C_35_H_69_O_10_N_0_P_1_Na_1_	2.5239	0.0118	0.8481	↓
MGDG (16:1/26:10)	GL	C_52_H_77_O_12_	1.4053	0.0455	1.0631	↑
DG (16:1/13:0)	GL	C_39_H_71_O_12_	1.2476	0.0279	1.0932	↑
DG (14:1e/18:1)	GL	C_35_H_66_O_4_Na_1_	1.7631	0.0426	0.8625	↓
TG (20:4e/9:0/20:3)	GL	C_58_H_104_O_5_N_2_	2.7350	0.0317	0.8371	↓
TG (20:3e/9:0/18:3)	GL	C_56_H_102_O_5_N_2_	1.7953	0.0396	0.9233	↓
TG (9:0/9:0/9:0)	GL	C_36_H_72_O_6_N_2_	1.2217	0.0050	1.0577	↑
TG (16:0/17:1/18:3)	GL	C_54_H_96_O_6_Li_1_	1.9556	0.0268	0.9232	↓
TG (14:0/11:2/20:2)	GL	C_54_H_100_O_6_N_2_	1.8311	0.0417	1.1041	↑
TG (18:2e/11:4/16:0)	GL	C_54_H_98_O_5_N_2_	2.7491	0.0266	0.8564	↓
TG (18:1e/11:3/22:6)	GL	C_60_H_102_O_5_N_2_	1.3292	0.0250	0.9286	↓

Note: SP. Sphingolipids; GP. Glycerophospholipids; GL. Glycerides; ↑. up; ↓. down.

## Data Availability

The original contributions presented in this study are included in the article/Supplementary Material. Further inquiries can be directed to the corresponding author(s).

## Supplementary Materials

The following supporting information can be downloaded at https://www.mdpi.com/article/10.3390/biotech14040099/s1, Figure S1: Chromatogram of the control group; Figure S2: Chromatogram of the NP + LY group; Figure S3: Chromatogram of the NP + LY-OE group.

## References

[1] Araujo F.G., Bauerfeldt G.F., Cid Y.P. (2018). Nonylphenol: Properties, legislation, toxicity and determination. An. Acad. Bras. Ciênc..

[2] Brandstaetter C., Fricko N., Rahimi M.J., Fellner J., Ecker-Lala W., Druzhinina I.S. (2022). The microbial metabolic activity on carbohydrates and polymers impact the biodegradability of landfilled solid waste. Biodegradation.

[3] Cheng C.Y., Wu C.Y., Wang C.H., Ding W.H. (2006). Determination and distribution characteristics of degradation products of nonylphenol polyethoxylates in the rivers of Taiwan. Chemosphere.

[4] Hong Y.J., Feng C.L., Xu D.Y., Wu F.C. (2023). Comprehensive Review on Environmental Biogeochemistry of Nonylphenol and Suggestions for the Management of Emerging Contaminants. Huan Jing Ke Xue.

[5] Yi C., Yang L., Yi R., Yu H., Zhang J., Nawaz M.I. (2022). Degradation of the nonylphenol aqueous solution by strong ionization discharge: Evaluation of degradation mechanism and the water toxicity of zebrafish. Water Sci. Technol..

[6] Milla S., Depiereux S., Kestemont P. (2011). The effects of estrogenic and androgenic endocrine disruptors on the immune system of fish: A review. Ecotoxicology.

[7] Ahmed S.A. (2000). The immune system as a potential target for environmental estrogens (endocrine disrupters): A new emerging field. Toxicology.

[8] Rogers J.A., Metz L., Yong V.W. (2013). Review: Endocrine disrupting chemicals and immune responses: A focus on bisphenol-A and its potential mechanisms. Mol. Immunol..

[9] Thompson P.A., Khatami M., Baglole C.J., Sun J., Harris S.A., Moon E.-Y., Al-Mulla F., Al-Temaimi R., Brown D.G., Colacci A.M. (2015). Environmental immune disruptors, inflammation and cancer risk. Carcinogenesis.

[10] Xie B., Qin J., Wang S., Li X., Sun H., Chen W. (2020). Adsorption of Phenol on Commercial Activated Carbons: Modelling and Interpretation. Int. J. Environ. Res. Public Health.

[11] Kuramitz H., Saitoh J., Hattori T., Tanaka S. (2002). Electrochemical removal of p-nonylphenol from dilute solutions using a carbon fiber anode. Water Res..

[12] Soares A., Guieysse B., Jefferson B., Cartmell E., Lester J.N. (2008). Nonylphenol in the environment: A critical review on occurrence, fate, toxicity and treatment in wastewaters. Environ. Int..

[13] Tanghe T., Dhooge W., Verstraete W. (1999). Isolation of a bacterial strain able to degrade branched nonylphenol. Appl. Environ. Microbiol..

[14] Fujii K., Urano N., Ushio H., Satomi M., Kimura S. (2001). *Sphingomonas cloacae* sp. nov., a nonylphenol-degrading bacterium isolated from wastewater of a sewage-treatment plant in Tokyo. Int. J. Syst. Evol. Microbiol..

[15] Gabriel F.L.P., Giger W., Guenther K., Kohler H.P.E. (2005). Differential degradation of nonylphenol isomers by *Sphingomonas xenophaga* Bayram. Appl. Environ. Microbiol..

[16] Corvini P.F.X., Schäffer A., Schlosser D. (2006). Microbial degradation of nonylphenol and other alkylphenols—Our evolving view. Appl. Microbiol. Biotechnol..

[17] Soares A., Murto M., Guieysse B., Mattiasson B. (2006). Biodegradation of nonylphenol in a continuous bioreactor at low temperatures and effects on the microbial population. Appl. Microbiol. Biotechnol..

[18] Gao Q.T., Wong Y.S., Tam N.F. (2011). Removal and biodegradation of nonylphenol by different *Chlorella* species. Mar. Pollut. Bull..

[19] Wang Z., Yang Y., Sun W., Dai Y., Xie S. (2015). Variation of nonylphenol-degrading gene abundance and bacterial community structure in bioaugmented sediment microcosm. Environ. Sci. Pollut. Res..

[20] Takeo M., Akizuki J., Kawasaki A., Negoro S. (2020). Degradation potential of the nonylphenol monooxygenase of *Sphingomonas* sp. NP5 for bisphenols and their structural analogs. Microorganisms.

[21] Elshaghabee F.M.F., Rokana N., Gulhane R.D., Sharma C., Panwar H. (2017). *Bacillus* as Potential Probiotics: Status, Concerns, and Future Perspectives. Front. Microbiol..

[22] Ishida R., Ueda K., Kitano T., Yamamoto T., Mizutani Y., Tsutsumi Y., Imoto K., Yamamori Y. (2019). Fatal community-acquired *Bacillus cereus* pneumonia in an immunocompetent adult man: A case report. BMC Infect. Dis..

[23] Kumar A., Chandra R. (2021). Biodegradation and toxicity reduction of pulp paper mill wastewater by isolated laccase producing *Bacillus cereus* AKRC03. Clean. Eng. Technol..

[24] Felshia S.C., Karthick N.A., Thilagam R., Chandralekha A., Raghavarao K.S.M.S., Gnanamani A. (2017). Efficacy of free and encapsulated *Bacillus lichenformis* strain SL10 on degradation of phenol: A comparative study of degradation kinetics. J. Environ. Manag..

[25] Kurihara T., Ueda M., Kamasawa N., Osumi M., Tanaka A. (1992). Physiological roles of acetoacetyl-CoA thiolase in n-alkane-utilizable yeast, *Candida tropicalis*: Possible contribution to alkane degradation and sterol biosynthesis. J. Biochem..

[26] Hashimoto Y., Ishigami K., Hassaninasab A., Kishi K., Kumano T., Kobayashi M. (2024). Curcumin degradation in a soil microorganism: Screening and characterization of a β-diketone hydrolase. J. Biol. Chem..

[27] Xie X., Liu J., Jiang Z., Li H., Ye M., Pan H., Zhu J., Song H. (2021). The conversion of the nutrient condition alter the phenol degradation pathway by *Rhodococcus biphenylivorans* B403: A comparative transcriptomic and proteomic approach. Environ. Sci. Pollut. Res..

[28] Lisboa N.S., Fahning C.S., Cotrim G., dos Anjos J.P., de Andrade J.B., Hatje V., da Rocha G.O. (2013). A simple and sensitive UFLC-fluorescence method for endocrine disrupters determination in marine waters. Talanta.

[29] Wu B., Xie Y., Xu S., Lv X., Yin H., Xiang J., Chen H., Wei F. (2020). Comprehensive Lipidomics Analysis Reveals the Effects of Different Omega-3 Polyunsaturated Fatty Acid-Rich Diets on Egg Yolk Lipids. J. Agric. Food Chem..

[30] Cunill J., Babot C., Santos L., Serrano J.C.E., Jové M., Martin-Garí M., Portero-Otín M. (2020). In Vivo Anti-Inflammatory Effects and Related Mechanisms of Processed Egg Yolk, a Potential Anti-Inflammaging Dietary Supplement. Nutrients.

[31] Li M., Li Q., Kang S., Cao X., Zheng Y., Wu J., Wu R., Shao J., Yang M., Yue X. (2020). Characterization and comparison of lipids in bovine colostrum and mature milk based on UHPLC-QTOF-MS lipidomics. Food Res. Int..

[32] Narayanan S., Zoong-Lwe Z.S., Gandhi N., Welti R., Fallen B., Smith J.R., Rustgi S. (2020). Comparative Lipidomic Analysis Reveals Heat Stress Responses of Two Soybean Genotypes Differing in Temperature Sensitivity. Plants.

[33] Xiang W., Shi R., Kang X., Zhang X., Chen P., Zhang L., Hou A., Wang R., Zhao Y., Zhao K. (2018). Monoacylglycerol lipase regulates cannabinoid receptor 2-dependent macrophage activation and cancer progression. Nat. Commun..

[34] Li J., Tang Q., Li Y., Fan Y.Y., Li F.H., Wu J.H., Min D., Li W.W., Lam P.K.S., Yu H.Q. (2020). Rediverting Electron Flux with an Engineered CRISPR-ddAsCpf1 System to Enhance the Pollutant Degradation Capacity of *Shewanella oneidensis*. Environ. Sci. Technol..

[35] Chopra N.K., Sondhi S. (2022). Cloning, expression and characterization of laccase from *Bacillus licheniformis* NS2324 in *E. coli* application in dye decolorization. Int. J. Biol. Macromol..

[36] Yadav D., Ranjan B., Mchunu N., Le Roes-Hill M., Kudanga T. (2021). Enhancing the expression of recombinant small laccase in *Pichia pastoris* by a double promoter system and application in antibiotics degradation. Folia Microbiol..

[37] Chai R., Guo J., Yang C., Zhu D., Li T., Yang W., Liu X., Chen X., Huang S., Wang H. (2025). Enhanced chemotaxis and degradation of nonylphenol in *Pseudoxanthomonas mexicana* via CRISPR-mediated receptor modification. Sci. Rep..

[38] Romantsov T., Guan Z., Wood J.M. (2009). Cardiolipin and the osmotic stress responses of bacteria. Biochim. Biophys. Acta BBA Biomembr..

[39] Carney O.S., Harris K., Santizo M., Silva V., Davis J., Lee K., Vishwanath S., Hamacher-Brady A., Vernon H.J. (2025). A review of disorders of cardiolipin metabolism: Pathophysiology, clinical presentation and future directions. Mol. Genet. Metab..

[40] Williams E.E. (1998). Membrane Lipids: What Membrane Physical Properties are Conserve during Physiochemically-Induced Membrane Restructuring?. Am. Zool..

[41] Chernomordik L. (1996). Non-bilayer lipids and biological fusion intermediates. Chem. Phys. Lipids.

[42] Sendecki A.M., Poyton M.F., Baxter A.J., Yang T., Cremer P.S. (2017). Supported Lipid Bilayers with Phosphatidylethanolamine as the Major Component. Langmuir.

[43] Murzyn K., Róg T., Pasenkiewicz-Gierula M. (2005). Phosphatidylethanolamine-phosphatidylglycerol bilayer as a model of the inner bacterial membrane. Biophys. J..

[44] Lee A.G. (2004). How lipids affect the activities of integral membrane proteins. Biochim. Biophys. Acta BBA Biomembr..

[45] Lee A.G. (2003). Lipid-protein interactions in biological membranes: A structural perspective. Biochim. Biophys. Acta BBA Biomembr..

[46] Bogdanov M., Dowhan W. (1995). Phosphatidylethanolamine is required for in vivo function of the membrane-associated lactose permease of *Escherichia coli*. J. Biol. Chem..

[47] Jimenez-Diaz L., Caballero A., Segura A., Rojo F.F. (2017). Pathways for the Degradation of Fatty Acids in Bacteria. Aerobic Utilization of Hydrocarbons, Oils and Lipids.

[48] Yang Y., Fang X., Yang R., Yu H., Jiang P., Sun B., Zhao Z. (2018). MiR-152 Regulates Apoptosis and Triglyceride Production in MECs via Targeting *ACAA2* and *HSD17B12* Genes. Sci. Rep..

