# Supplementary figures and images for: Enhancing Nonylphenol Biodegradation: The Role of Acetyl-CoA C-Acetyltransferase in Bacillus cereus
Source: BioTech (Basel). 2025 Dec 18;14(4):99. doi: 10.3390/biotech14040099 (PMC12730612; doi:10.3390/biotech14040099)

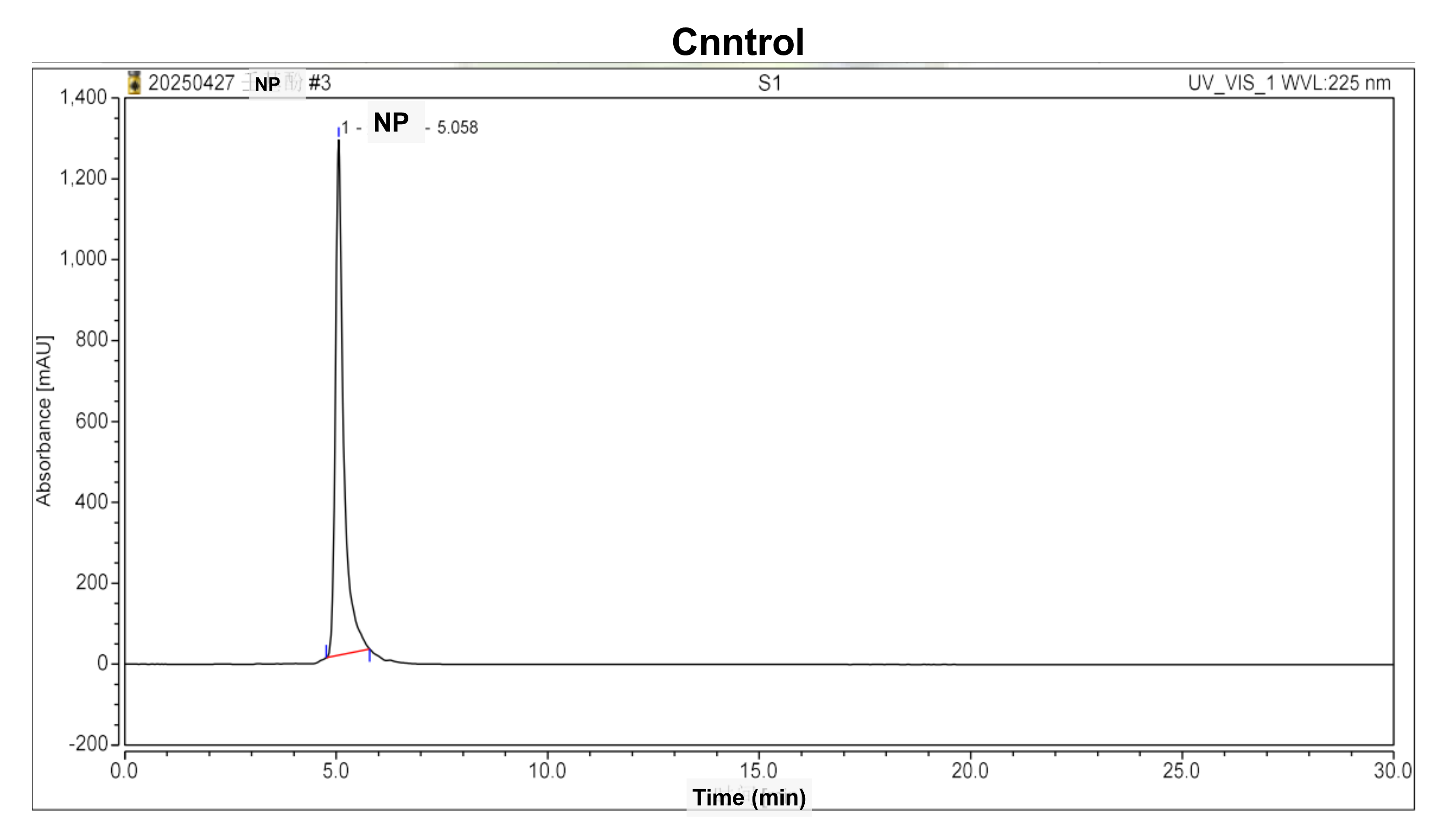

Supplement: Supplementary file 1 [file biotech-14-00099-s001.zip › S1.tif]

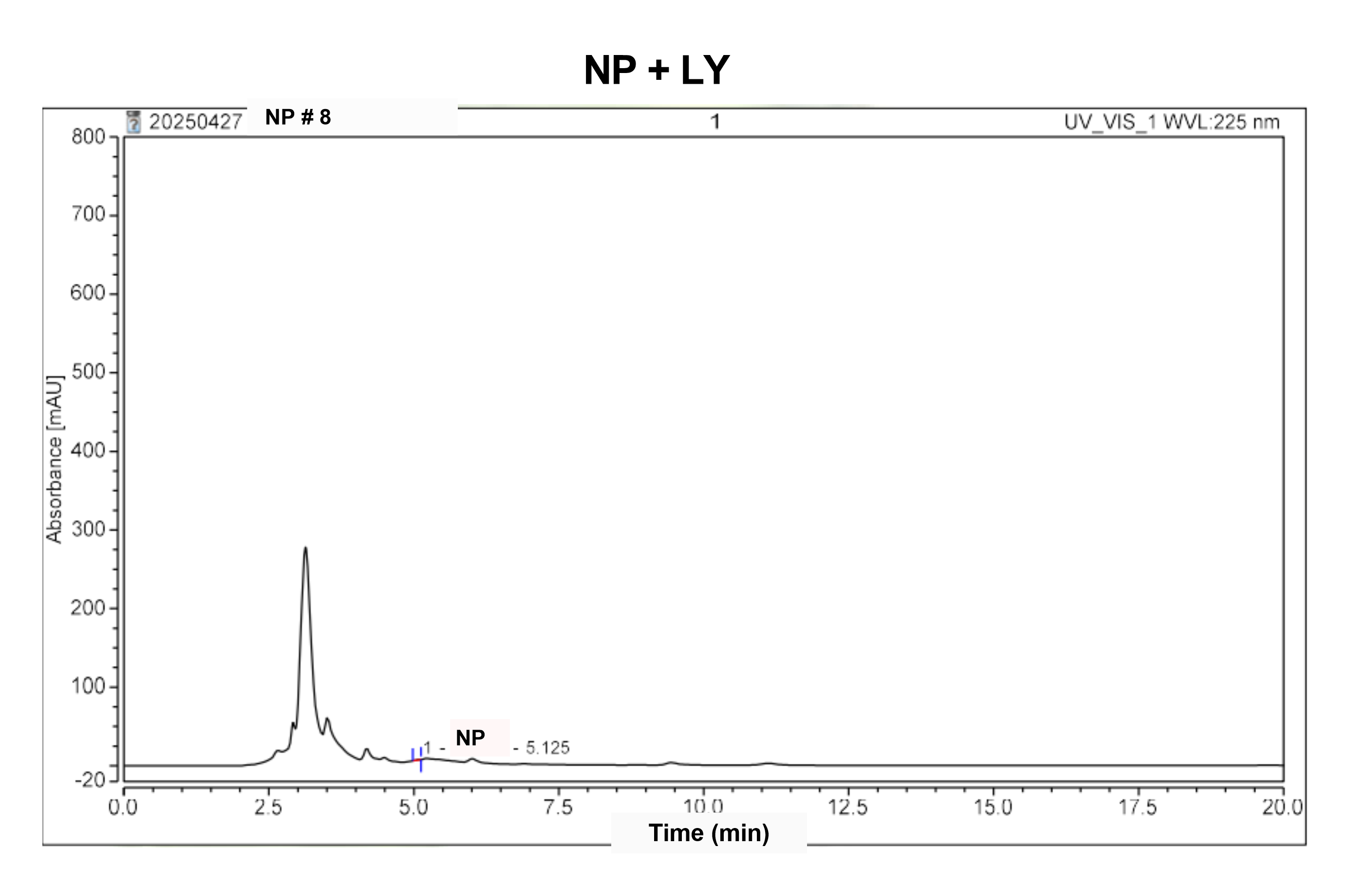

Supplement: Supplementary file 1 [file biotech-14-00099-s001.zip › S2.tif]

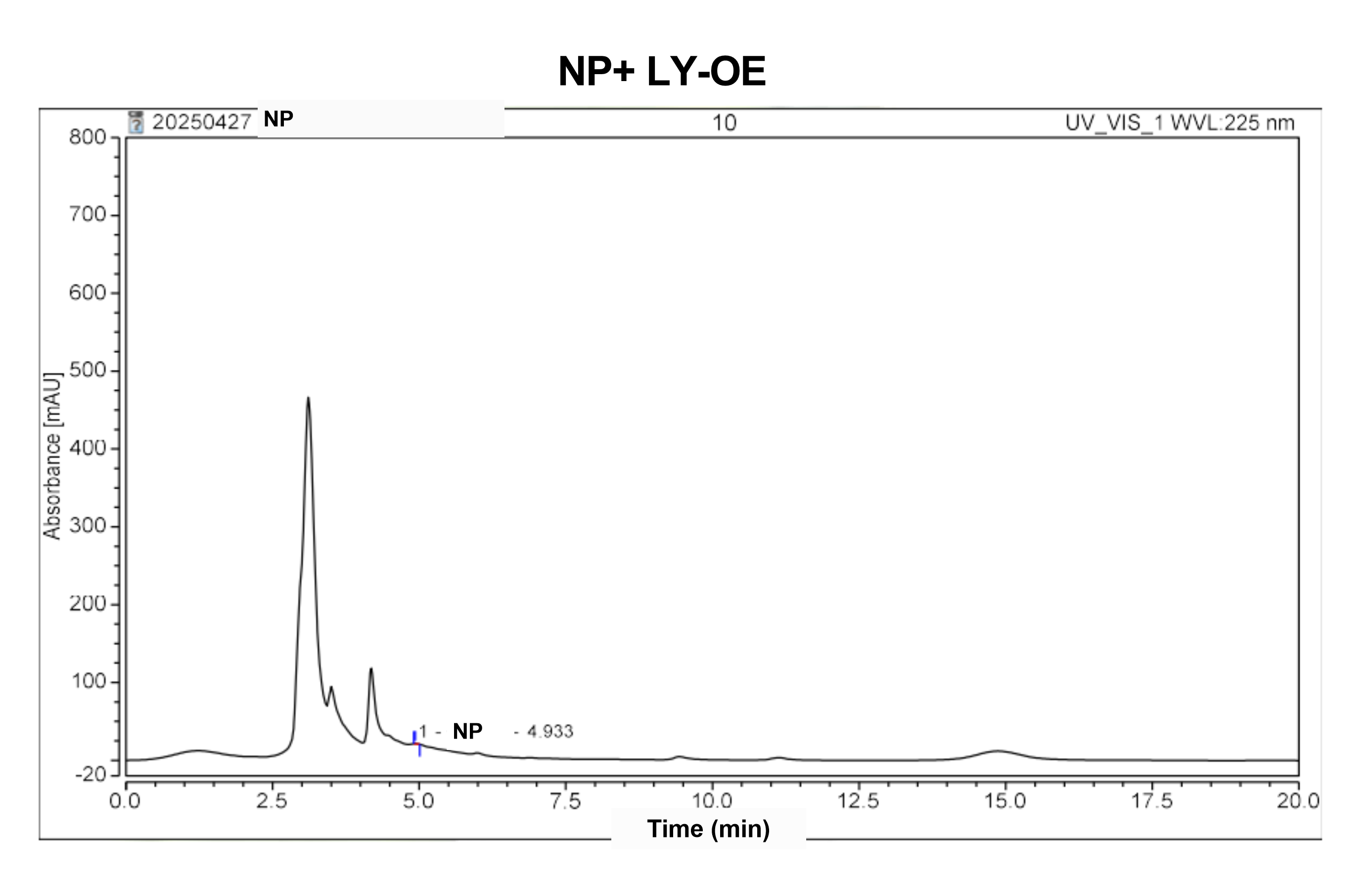

Supplement: Supplementary file 1 [file biotech-14-00099-s001.zip › S3.tif]
